# Genotype-by-environment interaction and genetic dissection of heartwood color in *Cryptomeria japonica* based on multiple common gardens and quantitative trait loci mapping

**DOI:** 10.1371/journal.pone.0270522

**Published:** 2022-07-06

**Authors:** Hideki Mori, Saneyoshi Ueno, Tokuko Ujino-Ihara, Takeshi Fujiwara, Kana Yamashita, Seiichi Kanetani, Ryota Endo, Asako Matsumoto, Kentaro Uchiyama, Takahiro Yoshida, Yoshimi Sakai, Yoshinari Moriguchi, Ryouichi Kusano, Yoshihiko Tsumura

**Affiliations:** 1 Forestry and Forest Products Research Institute, Tsukuba, Ibaraki, Japan; 2 Forest Bio-Research Center, Forestry and Forest Products Research Institute, Hitachi, Ibaraki, Japan; 3 Kyushu Research Center, Forestry and Forest Products Research Institute, Chuo, Kumamoto, Kumamoto, Japan; 4 Forestry Research Institute, Chiba Prefectural Agriculture and Forestry Research Center, Sammu, Chiba, Japan; 5 Graduate School of Science and Technology, Niigata University, Nishi-ku, Niigata, Japan; 6 Kumamoto Prefecture Forestry Research Center, Kumamoto, Japan; Federal University of Mato Grosso do Sul, BRAZIL

## Abstract

The heartwood color of a major plantation tree *Cryptomeria japonica* shows high variability among clones and cultivars, and brighter heartwood has higher value in the usage of non-laminated wood such as in traditional construction, which makes heartwood color an important trait in breeding of this species. However, the genetic basis of the interactions between genetics and the environment on heartwood color has been understudied while these are necessary for effective breeding programs in multiple environmental condition. The objectives of the present study were to evaluate the effects of genetics and environments on heartwood color and how they interact in contrasting environments, and to identify genomic regions controlling heartwood color in *C*. *japonica* across multiple environments. Heartwood color in terms of L*a*b* color space and spectral reflectance was measured in common gardens established in three contrasting sites. Quantitative trait loci (QTL) that affect heartwood color were identified using previously constructed highly saturated linkage maps. Results found that heartwood color was largely genetically controlled, and genotype-by-environment interaction explained one-third of the total genetic variance of heartwood color. The effect of the environment was small compared to the effect of genetics, whereas environmental effects largely varied among heartwood color traits. QTL analysis identified a large number of QTLs with small to moderate effects (phenotypic variation explained of 6.6% on average). Some of these QTLs were stably expressed in multiple environments or had pleiotropic effects on heartwood color and moisture content. These results indicated that genetic variation in phenotypic plasticity plays an important role in regulating heartwood color and that the identified QTLs would maximize the breeding efficiency of heartwood color in *C*. *japonica* in heterogeneous environments.

## Introduction

In forest trees, a number of studies on the inheritance and dissection of quantitative traits have focused on wood properties and growth (reviewed in [1]). In wood property traits, strength and quality-related traits, such as Young’s modulus and density, have been studied due to their importance in wood characteristics [2–4]. However, breeding of quantitative traits in tree species remains challenging because of long generation time of forest trees. Previous studies showed that selection for traits of interests based on marker-based genotypes which is known as marker-assisted selection (MAS) and selection of genotypes based on genomic breeding values derived from genome-wide DNA markers (genomic selection) substantially enhance breeding efficiency, and breeding approaches have attracted interest of plant breeders [5–7].

Quantitative traits, such as growth and wood properties, are characterized by genetic and environmental factors and interactions between genetics and environments [8–10]. Understanding genetic and environmental effect changes and genotype-by-environment interaction (genetic variation of phenotypic plasticity) in different environments is of primary importance in forest tree breeding and conservation under environmental changes, such as rapid climate changes and seed/seedling transfer via assisted migration [11]. Common garden experiments in multiple environments have provided important insights into the relative contribution of genetic and environmental factors on phenotypic traits, and genes or genomic regions that affect phenotypic traits have been identified using quantitative trait locus (QTL) and association mapping in forest trees (reviewed in [12]). Accumulation of the knowledge of QTLs would contribute to breeding of quantitative traits, however, the effect of QTLs could also change depending on the environmental conditions [13]. Thus, understanding the magnitude of these interactions across multiple environments is necessary for understanding the genetic architecture and improving the breeding efficiency of quantitative traits.

Wood color is one of the traits that most differentiate wood properties and is considered to enhance the value of the wood. In this context, wood color variations have been studied in various tree species [14–16]. However, studies on wood color trait dissection have remained scarce compared to other wood properties, such as wood strength. In some forest tree species, wood color is highly correlated with other wood properties, such as wood density and moisture content [14, 17–19]. For example, Rink and Phelps [14] reported a negative correlation between wood density and color (lightness) in *Juglans nigra*. Moya et al. [18] reported that wood density was negatively correlated with moisture content and b* (blue/yellow) color in teak (*Tectona grandis*). The esthetic value and large variability of heartwood color, and the strong correlations of heartwood color with other wood property traits described above imply that heartwood color is suitable for tree breeding and it may be possible to improve breeding efficiency of heartwood color through molecular breeding approaches.

*Cryptomeria japonica* (D. Don) is an allogamous, wind-pollinated, and evergreen conifer species [20]. This species is one of the most commercially important tree species characterized by rapid and straight growth with pleasant color and ease of handling [21] and has been used for a variety of purposes, such as house construction, shipbuilding, and making wooden barrels. This species could easily be vegetatively propagated, making it suitable for common garden experiments in multiple environments. Highly saturated linkage maps using a large number of genetic markers have been developed for this species [22, 23], which is necessary for genetic mapping studies [13, 24]. It is empirically well known that heartwood color of *C*. *japonica* could become dark or reddish in some cultivars [25–27]. For example, a previous study conducted in a single forest stand reported that the brightness of heartwood color (L*) largely differs between different clones of *C*. *japonica*, and moisture and potassium content are correlated with heartwood color [28]. In addition, darker heartwood color is less preferred for the usage of non-laminated wood such as in traditional construction [28]. However, the genetic basis of genotype-by-environment interaction and the genomic regions controlling heartwood color in *C*. *japonica* is largely unknown.

This study aimed to evaluate the relative effects of genetic and environmental factors and genotype-by-environment interaction on heartwood color in *C*. *japonica* and identify genomic regions that control the heartwood color of this species using multiple common garden experiments established in three contrasting environments. These common gardens have been used to identify QTLs of wood property and growth traits and the climate sensitivity traits of *C*. *japonica* in previous studies [13, 24]. This study further compared QTLs of heartwood color to previously reported QTLs of wood property, such as heartwood moisture content and density, to identify QTLs that could affect multiple heartwood property traits.

## Materials and methods

### Plant material, common garden experiment, and linkage map

The plant material, common garden experiment, and linkage map previously described by Mori et al. [13, 24] were used in this study. The mapping progeny comprised 139 genotypes from F1 plants, YI96 (female parent) and YI38 (male parent), were used as mapping populations in the present study. YI96 andYI38 were derived from local varieties of the Kyushu region (southern part of Japan): “Yabukuguri,” “Iwao,” and “Kumotooshi.” Previous studies have reported differences in the heartwood color of these cultivars [25, 29]. Significant differences in heartwood colors among these cultivars (*P* < 0.01, analysis of variance; for details, see S1 and S2 Tables) were also confirmed using wood samples of the cultivars obtained in a previous study [30]. YI96 was obtained by crossing “Yabukuguri” × “Iwao,” whereas YI38 was obtained by crossing “Yabukuguri” × “Kumotooshi.” Common garden experiments were established for the 139 genotypes, with three clonal replicates in three different environments in Japan: Ibaraki site (36°11′4.66″N, 140°13′1.80″E), Chiba site (35°20′52.55″N, 140°1′47.92″E), and Kumamoto site (32°41′58.36″N, 130°45′17.64″E); the experiments consisted of three clonal replicates with a randomized block design with each tree planted at an interval of 1.8 m. Local varieties ‘Yanase’, ‘Sanbu’, and ‘Shakain’ were planted at the surrounding edges of the study site in Ibaraki, Chiba, and Kumamoto, respectively, to minimize the edge effect [13]. Three to five soil samples from each site were collected for soil analysis to evaluate water content, total carbon (g/kg), total nitrogen (g/kg), pH (H_2_O, KCl), and exchangeable cations (Ca, Mg, K, Na; cmol(+)/kg) in 2017 (S3 Table). Two linkage maps based on 858 genetic markers (YI96: heterozygous; YI38: homozygous) and 916 genetic markers (YI38: heterozygous; YI96: homozygous) were used to construct the YI96 and YI38 linkage maps, respectively. Genetic markers with large numbers of missing values (>1%) were excluded. The average marker distance between adjacent markers in the YI96 and YI38 linkage maps was 1.47 and 1.56 cM, respectively.

### Measurement of heartwood color

Wood samples from 40 to 80 cm above ground were collected from each mapping progeny to measure heartwood color. Unedged boards (5 cm) with pith were sawn using a bandsaw and dried indoors for >1 year to attain the air-dried state. The quartersawn faces of the wood samples were planed with a hand-feed planer before heartwood color measurement. Heartwood color was measured as L*a*b* color space (CIELAB; lightness L*, green to red hues a*, and blue to yellow hues b*) and spectral reflectance (400–700 nm with 20 nm interval), which are commonly used to assess wood color in various tree species, including *C*. *japonica* [31, 32]. Heartwood color traits were measured using a spectrophotometer NF333 (Nippon Denshoku Industries, Tokyo, Japan), with a measurement area and a light source of φ8 mm and D65/10°, respectively. Heartwood color measurement was done at five equal distance points at the center and the edges (left and right) of the heartwood in a longitudinal direction, resulting in 15 measurement points per wood sample. When the heartwood width of the sample was narrow, the center of the heartwood was measured, resulting in five measurement points for those samples. For each trait and site, the observed values were averaged for each sample, and genotypes with two or more replicates per site were retained for subsequent analyses.

### Phenotypic data analysis

Heartwood color can be spatially biased based on microscale environmental conditions within sites. For example, the heartwood color of *C*. *japonica* is affected by soil potassium content [33]. Thus, Moran’s *I* test was conducted to investigate the level of spatial autocorrelation for each trait using the R package “spdep” [34]. A linear model for the block design (with block and genotype as fixed effects) was fitted for each site and trait, and the residuals were used for Moran’s *I* tests. Moran’s *I* statistic ranges from −1 (perfectly dispersed) to 1 (perfectly aggregated). The significance of spatial autocorrelation was tested under the assumption of spatial randomness.

The genotypic means (best linear unbiased estimators) for each site and trait were predicted using the linear model for the block design. When there was a significant positive spatial autocorrelation with Moran’s *I* test for a given trait within a site (*P* < 0.05; S4 Table), the genotypic means were predicted using the model for the block design with additional spatial autocorrelation terms (penalized spline curves; P-splines) using the R package “SpATS” [35]. The predicted genotypic means for each site were used for subsequent QTL analyses and the correlation matrix (see below).

The contribution of genetic and environmental effects to the total variability of heartwood color traits was estimated using variance components analyses. Each trait, genotype, site, block (nested within sites), and interactions between genotypes and sites were used as random variables to estimate variance components and 95% confidence intervals (CIs). Variance component analyses were performed using the “anovaVCA” function of the R package “VCA.” To further understand the environmental effects of three sites on heartwood color traits, the difference in phenotypic values among the sites for each trait was tested using the post-hoc analysis of Tukey’s all-pairs comparison. Phenotypic data analysis was performed using R version 4.1.1 [36], and multiple comparison tests were performed using the R package “multcomp” [37]. Correlation coefficients (Pearson’s *r*) among the genotypic means of the heartwood color traits were obtained for each site, and the level of significance of the correlations was tested with *P*-values calculated by the t-distribution using the R package “Hmisc.” Correlations among heartwood color traits were visualized with heatmaps of the correlation matrix for each site using the R package “corrplot” (Fig 2) and with correlation network plots using the R package “corrr” (S1 Appendix).

### QTL analysis

The methodology of QTL analysis conducted in this study was fully described by Goto et al. [38]. QTL analysis was conducted using hierarchical linear models with a regularized horseshoe prior distribution. One marker from a given pair of markers that were closely located (<1 cM) and showed a high correlation (r > 0.7) with other markers was removed to reduce collinearity and redundancy among variables. The posterior distribution was estimated using four chains with 5000 iterations after the first 3000 iterations as burn-in using the R package “rstanarm” [39]. The convergence of the chains was confirmed using the Gelman-Rubin statistic ($\hat{R}$ < 1.1). The global shrinkage parameter of the regularized horseshoe prior was set using the equation proposed by Piironen and Vehtari [40]; $\frac{p0}{D-p0}\frac{1}{\sqrt{n}}$, where *p0* is the expected number of relevant variables, *D* is the number of variables, and *n* is the number of observations. *p0* was set to 5 for all traits, and the sensitivity of the *p0* value was confirmed by ranging *p0* from 1 to 9 (S5 Table). The default values of the regularized horseshoe prior in the R package “rstanarm” were used for the remaining parameters.

The marginals of the posterior distribution of marker effects may be biased due to the high correlation among variables. Thus, a model selection method (projection-predictive variable selection) [40] was applied to the model. This method uses a model with the best predictive power (a reference model) to find a simpler model with similar predictions as the reference model (predictive projection). The linear model described above was used as the reference model. Model selection was done when variables in the reference model contained significantly more information than the null model, which was tested by leave-one-out cross-validation [41]. When the marginals of the marker effects in the selected model did not contain zero in the 95% and 80% confidence intervals (CIs), the markers were defined as significant and suggestive QTLs, respectively. The contribution of each QTL was estimated by the coefficient of determination (R^2^) of the simple regression, and the contribution was defined as the phenotypic variation explained (PVE) for a QTL. Because the resolution of QTL positions depends on the size of the mapping progeny, QTLs that were closely located should be considered the same QTL. In this study, QTLs located within 5 cM were defined as the same QTL. QTL analysis and model selection were done using R version 4.1.1 [36]. The leave-one-out cross-validation procedure was performed using the R package “loo” [41], and the model selection method was done using the R package “projpred” [42]. Because L*a*b* color space and spectral reflectance were highly correlated (see Results), QTLs were shown only for L*, a*, and b* in the QTL analysis results to avoid complexity. The QTL analysis results of spectral reflectance can be found in the (S2 and S3 Appendices; S6–S8 Tables).

## Results

### Heartwood color traits

**Fig 1:**
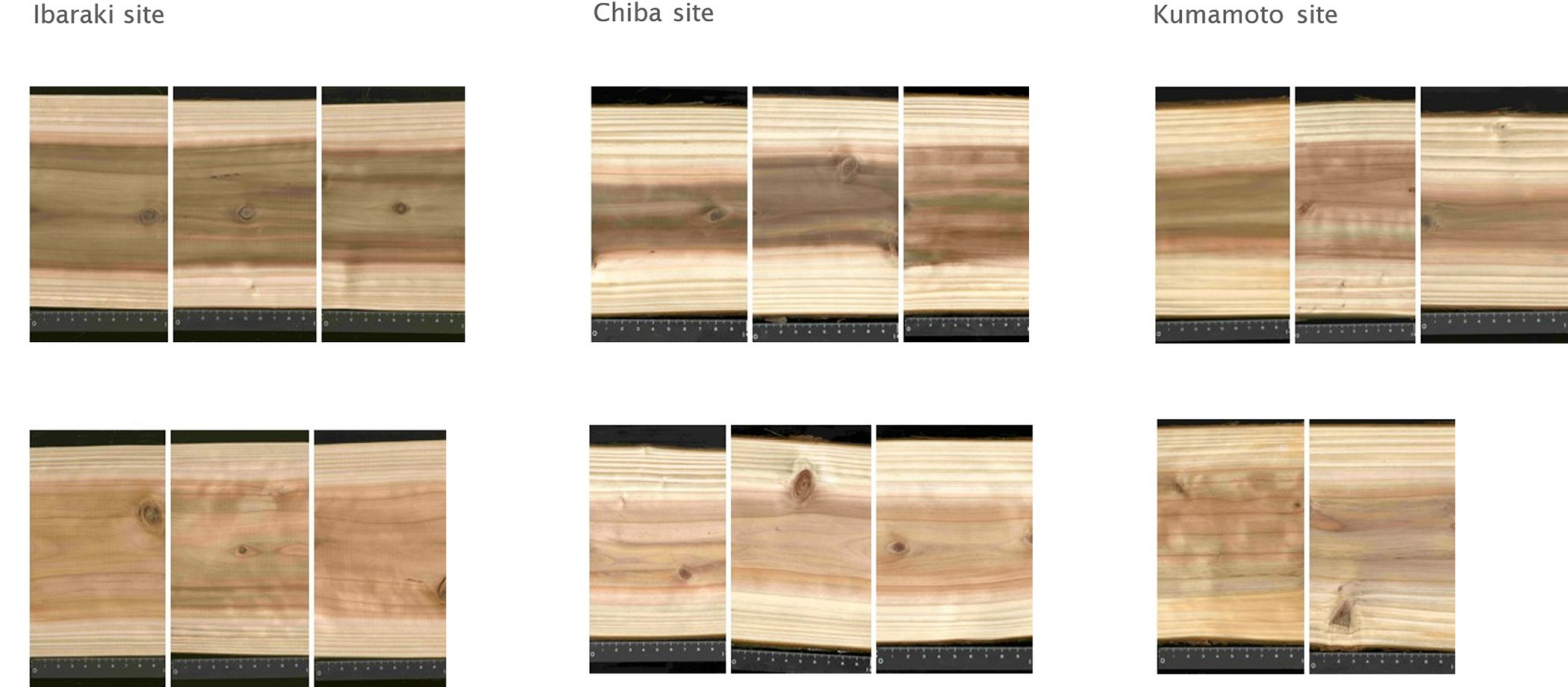
Scanned images of wood samples of a genotype (with two or more replicates per site) with the lowest L* (i.e., darkest heartwood color; top row) and a genotype with the highest L* (i.e., brightest heartwood color; bottom row) in the three sites.

Heartwood color traits (L*a*b* color space and spectral reflectance) of mapping progenies in the three sites are summarized in Table 1, and the sample scanned images of the sample clones (genotypes) with the highest and lowest L* are shown in Fig 1. Significant spatial autocorrelation was detected for all heartwood color traits in the Kumamoto site and a* and b* in the Ibaraki site, whereas no significant spatial autocorrelation was found for heartwood color traits in the Chiba site (S4 Table). L* in the Ibaraki site was significantly smaller (i.e., darker) than Chiba and Kumamoto sites (*P* < 0.05; Table 1). a* in the Chiba site was significantly larger (i.e., more reddish) than Ibaraki and Kumamoto sites (*P* < 0.05). This tendency was also observed in significantly higher spectral reflectance in longer wavelengths (640–700 nm) in the Chiba site (*P* < 0.05). b* in the Kumamoto site was significantly smaller (i.e., more blueish) than the other two sites (*P* < 0.05). This tendency was also observed in significantly higher spectral reflectance in shorter wavelengths (440–500 nm) in the Kumamoto site (*P* < 0.05).

**Table 1:** Summary of the heartwood color traits in the three common gardens.

Trait	Ibaraki site					Chiba site					Kumamoto site				
	Mean	SD^a^	CV^b^	Min^c^	Max^d^	Mean	SD	CV	Min	Max	Mean	SD	CV	Min	Max
L*a*b* color space															
L*	57.66a	3.87	0.067	47.75	72.19	58.23b	4.68	0.080	45.49	73.14	58.49b	3.55	0.061	47.13	67.87
a*	11.58b	1.12	0.097	8.50	15.98	12.46c	1.20	0.096	9.30	17.61	11.27a	1.34	0.119	7.91	16.34
b*	20.75b	1.37	0.066	16.45	24.30	20.80b	1.33	0.064	16.78	23.73	20.14a	1.10	0.055	16.39	23.91
Spectral reflectance															
400 nm	12.70a	1.87	0.147	8.37	22.36	13.44b	2.48	0.185	7.88	23.23	13.50b	1.98	0.147	8.28	20.45
420 nm	13.48a	2.08	0.154	8.74	24.20	14.09b	2.74	0.194	8.13	25.51	14.33b	2.20	0.154	8.50	22.00
440 nm	14.32a	2.30	0.161	9.16	26.02	14.86b	3.01	0.203	8.46	27.83	15.23c	2.41	0.158	8.79	23.49
460 nm	15.44a	2.53	0.164	9.81	28.18	15.97b	3.30	0.207	8.99	30.46	16.43c	2.64	0.161	9.36	25.38
480 nm	16.85a	2.72	0.161	10.72	30.32	17.41b	3.49	0.200	9.90	32.39	17.88c	2.79	0.156	10.31	27.60
500 nm	19.21a	2.97	0.155	11.99	33.40	19.75b	3.68	0.186	11.48	34.39	20.19c	2.94	0.146	12.05	30.22
520 nm	20.97a	3.29	0.157	12.84	36.11	21.39b	4.05	0.189	12.12	37.83	21.79b	3.17	0.145	12.97	31.92
540 nm	22.33a	3.58	0.160	13.65	38.41	22.66a	4.39	0.194	12.51	40.31	23.18b	3.42	0.148	13.66	34.04
560 nm	26.58a	4.24	0.160	16.65	45.37	27.18b	5.14	0.189	15.02	46.12	27.59b	3.97	0.144	16.47	39.79
580 nm	29.87a	4.76	0.159	19.39	50.75	30.84b	5.85	0.190	16.95	52.00	30.78b	4.40	0.143	18.44	43.07
600 nm	33.65a	5.25	0.156	22.39	56.04	34.89b	6.43	0.184	19.67	57.48	34.46b	4.81	0.140	20.98	47.61
620 nm	38.27a	5.79	0.151	26.17	62.10	39.93b	7.06	0.177	23.33	63.78	38.99b	5.27	0.135	24.06	53.26
640 nm	41.86a	6.17	0.147	28.93	66.26	43.80b	7.49	0.171	26.13	67.98	42.49a	5.59	0.132	26.45	57.06
660 nm	44.34a	6.38	0.144	30.79	68.27	46.27b	7.67	0.166	27.92	69.67	44.80a	5.77	0.129	28.26	59.82
680 nm	45.43a	6.51	0.143	31.63	69.62	47.46b	7.83	0.165	28.72	71.06	45.83a	5.87	0.128	28.92	61.14
700 nm	45.90a	6.56	0.143	31.87	70.26	48.02b	7.88	0.164	29.20	71.76	46.34a	5.92	0.128	29.30	61.71

For each trait, different letters beside the numerals show significant differences between sites according to Tukey’s post hoc test (p < 0.05).

^a^ standard deviation

^b^ coefficient of variation

^c^ minimum value

^d^ maximum value.

**Fig 2:**
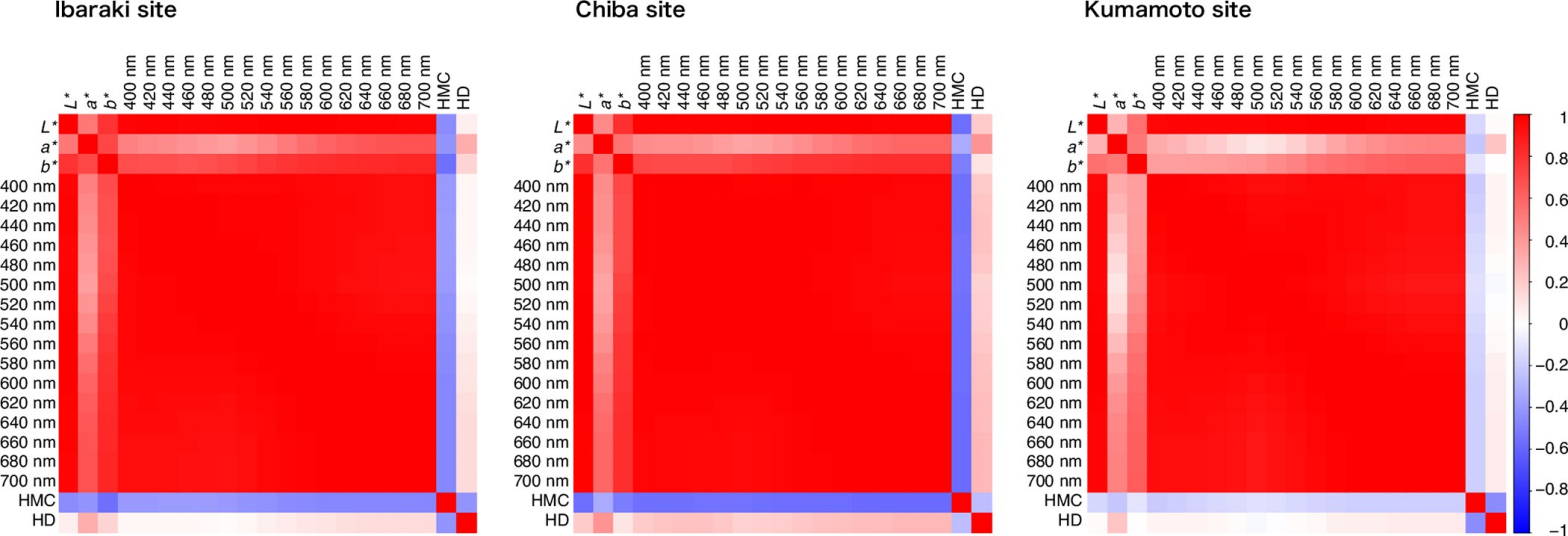
Correlation heatmap for heartwood color traits (L*a*b* color space and spectral reflectance) and heartwood property traits (density and moisture content).

Correlations among heartwood colors and properties (moisture content and density) are summarized in Fig 2. All heartwood colors were significantly positively correlated (*P <* 0.001; S9 Table); L* and spectral reflectance had a strong and significant positive correlation (*r* > 0.96; *P* < 0.001), whereas relatively small to moderate significant positive correlations were found among L*, a*, and b* (0.801 > *r* > 0.297; *P* < 0.001) at the three study sites. Heartwood moisture content was significantly negatively correlated with all heartwood color traits at the Chiba and Ibaraki sites (−0.326 > *r* > −0.576; *P* < 0.001; S9 Table) and with some heartwood color traits at the Kumamoto site (−0.180 > *r* > −0.220; S9 Table). Heartwood density was significantly positively correlated with a* at the three sites (0.417 > *r* > 0.220; *P* < 0.01; S9 Table).

### Proportion of the variability of heartwood color traits explained by genotype and environment

The proportion of variance components of genotype, site, block, and interactions between genotypes and sites in heartwood color traits are shown in Table 2. All heartwood color traits were significantly different between genotypes and interactions between genotypes and sites in terms of 95% CI (Table 2). Genotypes, sites, and their interaction explained 14.2%–33.9%, 1.7%–14.6%, and 7.9%–15.2% of the total variability of heartwood color traits, respectively. Genotypes and environments (site and block) explained the largest and smallest variance among variables in all heartwood color traits. The variance components of genotypes were larger (>33.1%) in longer wavelengths (>580 nm) and L* than a* (14.2%) and b* (18.5%). The variance components of sites were largest in a* (14.6%) and were small (1.7%–2.9%) in L* and intermedium to long wavelengths (520–700 nm). The variance components of interactions between genotypes and sites were larger in a* (13.0%) and b* (15.2%) than L* and spectral reflectance (7.9%–12.8%).

**Table 2:** Proportion of the variations in heartwood color traits explained by genotype, environment, and the interaction between genotype and environment (i.e., plasticity).

Trait	Variance components (%)				
	Genotype	Site	Block	Interaction between Genotype and Site	Residuals
L*a*b* color space					
L*	33.4*	2.4	1.0	10.4*	52.8*
a*	14.2*	14.6	3.3	13.0*	54.9*
b*	18.5*	3.5	1.6	15.2*	61.2*
Spectral reflectance					
400 nm	30.6*	6.2	2.3	7.9*	53.0*
420 nm	31.2*	5.4	1.9	8.0*	53.5*
440 nm	31.3*	5.2	1.7	8.2*	53.6*
460 nm	31.1*	5.1	1.6	8.3*	53.9*
480 nm	30.7*	5.1	1.5	8.4*	54.3*
500 nm	30.7*	4.4	1.3	8.6*	55.0*
520 nm	31.6*	2.9	1.2	9.2*	55.1*
540 nm	32.2*	2.8	1.0	9.3*	54.7*
560 nm	32.7*	3.0	0.9	9.9*	53.5*
580 nm	33.1*	2.1	0.9	11.1*	52.8*
600 nm	33.4*	1.7	1.0	11.6*	52.3*
620 nm	33.6*	1.9	0.9	12.0*	51.6*
640 nm	33.7*	2.0	0.8	12.3*	51.2*
660 nm	34.0*	1.8	0.7	12.7*	50.8*
680 nm	33.9*	1.9	0.6	12.8*	50.8*
700 nm	33.9*	2.1	0.6	12.7*	50.7*

Asterisks represent the significance of the variables based on 95% CI of the estimated variance components.

### QTL analysis

**Fig 3:**
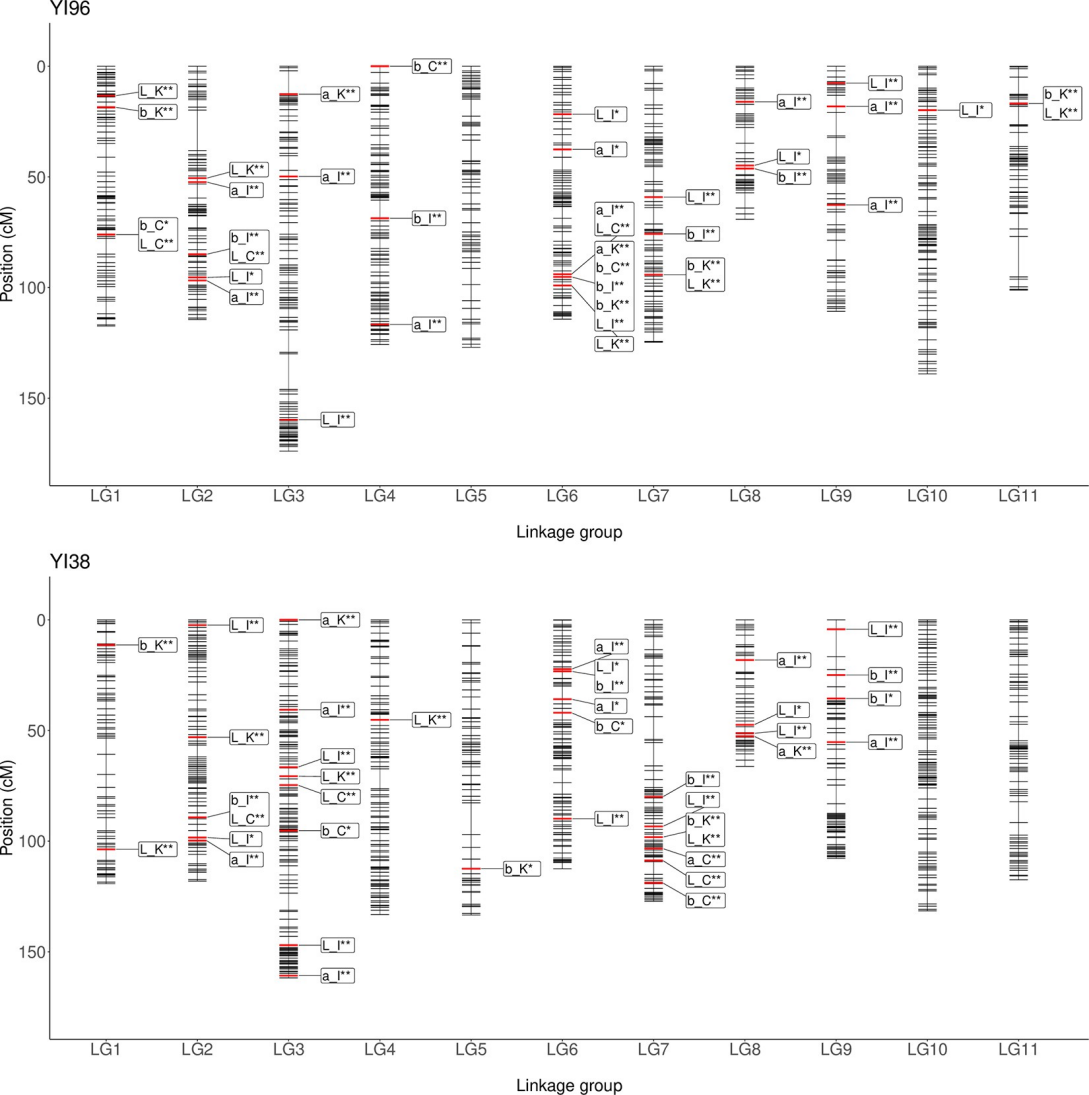
Linkage maps with QTLs of heartwood color traits (L*, a*, and b*). Short horizontal bars represent the position of the markers, and the red horizontal bars represent QTLs. QTLs were labeled using trait_site. Abbreviations: L, L*; a, a*; b, b*; I, Ibaraki; C, Chiba; K, Kumamoto. Level of significance of QTLs: **, 95% CI; *, 80% CI.

QTL analysis identified 64 significant QTLs and 12 suggestive QTLs for heartwood color traits of L*a* b* color space in the three sites (Table 3; Fig 3). The number of QTLs identified for L*, a*, and b* were 34, 21, and 23, respectively (S4 Appendix). The number of QTLs identified for L*a*b* color space in Ibaraki, Chiba, and Kumamoto sites were 44, 13, 21, respectively. One stable QTL (a QTL identified in all sites) was identified for b* (S7 Table). Three QTLs were identified for L*a*b* color space in the two sites: one QTL was identified each in Ibaraki and Chiba sites, Ibaraki and Kumamoto sites, and Chiba and Kumamoto sites. Some of the identified QTLs were clustered in genomic regions, such as 94.0 to 99.1 cM in LG06 of the YI96 map (Fig 3). In addition, 6 of 13 QTLs with PVE > 10 were located in LG09. Two QTLs of L* were considered as the same QTLs identified for heartwood moisture content in a previous study [13] (S8 Table).

**Table 3:** List of QTLs identified for heartwood color traits (L*, a*, and b*) in the three sites.

Trait	Site	Map	LG	Marker	Position	Significance	PVE	Stableness^a^
L*	Ibaraki	YI38	LG02	m_0928	2.412	**	5.1	unique
L*	Ibaraki	YI38	LG02	m_0998	98.363	*	6.4	unique
L*	Ibaraki	YI38	LG03	m_1069	66.575	**	4.4	unique
L*	Ibaraki	YI38	LG03	m_1126	146.995	**	8.2	unique
L*	Ibaraki	YI38	LG06	m_1332	23.305	*	2.9	unique
L*	Ibaraki	YI38	LG06	m_1382	89.862	**	1.5	unique
L*	Ibaraki	YI38	LG07	m_1455	93.335	**	6.0	unique
L*	Ibaraki	YI38	LG08	m_1521	47.435	*	6.0	unique
L*	Ibaraki	YI38	LG08	m_1524	51.286	**	2.6	unique
L*	Ibaraki	YI38	LG09	m_1540	4.265	**	12.1	unique
L*	Chiba	YI38	LG02	m_0994	89.243	**	6.0	unique
L*	Chiba	YI38	LG03	m_1073	74.668	**	3.7	†
L*	Chiba	YI38	LG07	m_1473	108.725	**	4.6	unique
L*	Kumamoto	YI38	LG01	m_0912	103.673	**	7.1	unique
L*	Kumamoto	YI38	LG02	m_0963	53.036	**	7.2	unique
L*	Kumamoto	YI38	LG03	m_1071	70.654	**	8.4	†
L*	Kumamoto	YI38	LG04	m_1181	45.196	**	3.8	unique
L*	Kumamoto	YI38	LG07	m_1459	98.171	**	6.6	unique
a*	Ibaraki	YI38	LG02	m_0999	99.742	**	7.5	unique
a*	Ibaraki	YI38	LG03	m_1044	40.59	**	2.8	unique
a*	Ibaraki	YI38	LG03	m_1152	160.764	**	0.5	unique
a*	Ibaraki	YI38	LG06	m_1331	22.273	**	3.8	unique
a*	Ibaraki	YI38	LG06	m_1340	35.877	*	7.0	unique
a*	Ibaraki	YI38	LG08	m_1504	18.176	**	3.2	unique
a*	Ibaraki	YI38	LG09	m_1560	55.283	**	15.3	unique
a*	Chiba	YI38	LG07	m_1466	103.352	**	11.6	unique
a*	Kumamoto	YI38	LG03	m_1015	0	**	6.2	unique
a*	Kumamoto	YI38	LG08	m_1525	52.586	**	8.8	unique
b*	Ibaraki	YI38	LG02	m_0994	89.243	**	7.2	unique
b*	Ibaraki	YI38	LG06	m_1332	23.305	**	5.0	unique
b*	Ibaraki	YI38	LG07	m_1443	79.962	**	2.9	unique
b*	Ibaraki	YI38	LG09	m_1543	24.951	**	17.4	unique
b*	Ibaraki	YI38	LG09	m_1545	35.544	*	18	unique
b*	Chiba	YI38	LG03	m_1098	95.216	*	4.9	unique
b*	Chiba	YI38	LG06	m_1343	41.999	*	6.2	unique
b*	Chiba	YI38	LG07	m_1479	118.883	**	8.3	unique
b*	Kumamoto	YI38	LG01	m_0866	11.352	**	7.5	unique
b*	Kumamoto	YI38	LG05	m_1296	112.45	*	4.9	unique
b*	Kumamoto	YI38	LG07	m_1459	98.171	**	5.7	unique
L*	Ibaraki	YI96	LG02	m_0141	95.414	*	6.4	unique
L*	Ibaraki	YI96	LG03	m_0241	159.694	**	8.2	unique
L*	Ibaraki	YI96	LG06	m_0446	21.71	*	2.9	unique
L*	Ibaraki	YI96	LG06	m_0497	95.18	**	10.1	†
L*	Ibaraki	YI96	LG07	m_0547	59.135	**	8.8	unique
L*	Ibaraki	YI96	LG08	m_0623	44.867	*	6.0	unique
L*	Ibaraki	YI96	LG09	m_0648	7.65	**	12.1	unique
L*	Ibaraki	YI96	LG10	m_0727	19.838	*	1.7	unique
L*	Chiba	YI96	LG01	m_0057	76.028	**	9.3	unique
L*	Chiba	YI96	LG02	m_0132	84.972	**	6.0	unique
L*	Chiba	YI96	LG06	m_0495	93.968	**	7.5	†
L*	Kumamoto	YI96	LG01	m_0017	13.354	**	10.4	unique
L*	Kumamoto	YI96	LG02	m_0108	50.59	**	7.2	unique
L*	Kumamoto	YI96	LG06	m_0500	99.071	**	10.2	unique
L*	Kumamoto	YI96	LG07	m_0571	94.196	**	6.6	unique
L*	Kumamoto	YI96	LG11	m_0815	16.817	**	11.6	unique
a*	Ibaraki	YI96	LG02	m_0109	52.332	**	3.8	unique
a*	Ibaraki	YI96	LG02	m_0142	96.798	**	7.5	unique
a*	Ibaraki	YI96	LG03	m_0184	49.796	**	2.8	unique
a*	Ibaraki	YI96	LG04	m_0356	116.684	**	5.6	unique
a*	Ibaraki	YI96	LG06	m_0453	37.607	*	7.0	unique
a*	Ibaraki	YI96	LG06	m_0495	93.968	**	9.1	†
a*	Ibaraki	YI96	LG08	m_0609	16.068	**	3.2	unique
a*	Ibaraki	YI96	LG09	m_0655	18.153	**	8.7	unique
a*	Ibaraki	YI96	LG09	m_0678	62.676	**	15.3	unique
a*	Kumamoto	YI96	LG03	m_0160	12.63	**	6.2	unique
a*	Kumamoto	YI96	LG06	m_0497	95.18	**	10.2	†
b*	Ibaraki	YI96	LG02	m_0132	84.972	**	7.2	unique
b*	Ibaraki	YI96	LG04	m_0315	68.707	**	0.8	unique
b*	Ibaraki	YI96	LG06	m_0497	95.18	**	12.3	††
b*	Ibaraki	YI96	LG07	m_0557	75.759	**	2.9	unique
b*	Ibaraki	YI96	LG08	m_0624	46.253	**	7.2	unique
b*	Chiba	YI96	LG01	m_0057	76.028	*	7.1	unique
b*	Chiba	YI96	LG04	m_0268	0	**	7.6	unique
b*	Chiba	YI96	LG06	m_0497	95.18	**	6.4	††
b*	Kumamoto	YI96	LG01	m_0020	18.52	**	7.5	unique
b*	Kumamoto	YI96	LG06	m_0497	95.18	**	8.6	††
b*	Kumamoto	YI96	LG07	m_0571	94.196	**	5.7	unique
b*	Kumamoto	YI96	LG11	m_0815	16.817	**	9.5	unique

LG, linkage groups; PVE, phenotypic variation explained (%). Level of significance of QTLs: **, 95% CI; *, 80% CI (see Methods for details).

^a^ ††: QTL identified in three sites. †: QTL identified in two sites. unique: QTL identified in one site.

## Discussion

### Heartwood color traits

Heartwood color traits observed for mapping progenies were comparable to previous reports on cultivars used in this study (i.e., Yabukuguri, Iwao, and Kumotooshi cultivars). For example, Kawazumi et al. [25] reported that L* of Kumotooshi and Yabukuguri cultivars were 56.8 ± 7.2 and 69.3 ± 3.0, respectively. Tsushima et al. [29] investigated the heartwood color of *C*. *japonica* cultivars in different initial spacings and reported that L*, a*, and b* were 65.2 to 69.4, 5.3 to 8.0, and 19.8 in Iwao cultivar and 72.2 to 73.7, 8.3 to 9.4, and 22.6 to 23.8 in Yabukuguri cultivar, respectively. Observations in previous reports and this study (S1 Table) on the three cultivars are mostly within the range of the observed values of L*, a*, and b* of mapping progenies. These findings indicate that heartwood color in this species is genetically preserved in various conditions (e.g., tree age, environments), which would be important for the improvement of this traits in tree-breeding programs.

Heartwood color traits, especially for L*, were negatively correlated with the heartwood moisture content, indicating that heartwood with higher moisture content is darker in color in *C*. *japonica* mapping progenies. This tendency is empirically known and has also been reported in previous studies [25, 29]. For example, Tsushima et al. [29] reported that the average values of L* and green moisture content of heartwood of Yabukuguri cultivar were 72.2, 72.6, and 73.7 and 168%, 163%, and 145% in the initial spacing of 1500, 3000, and 5000 trees/ha, respectively. In addition, Sugimoto et al. [43] reported that the lightness of *C*. *japonica* wood depends on the density and the amount of reflection in the layer, especially at long wavelengths. This study also found a weak but significant positive correlation between reflectance at long wavelengths (>600 nm) and heartwood density at Chiba site but almost no significant correlation between reflectance at short to intermediate wavelengths (400–600 nm) and density. These results implied that moisture content is more related than density with heartwood color in *C*. *japonica*. Furthermore, these suggested that heartwood color is not only important in terms of esthetic value but also a valuable indicator that represent wood moisture which could affect drying processes of the wood [28].

### Relative contribution of genotype, environment, and genotype-by-environment interaction on heartwood color

Although the effects of genotype and genotype-by-environment interaction on heartwood color traits were all significant, the relative contribution of these variables varied largely on heartwood color traits of mapping progenies. Genetic effects alone explained 30.7% on average of the total variance of heartwood color traits, whereas environmental effects (i.e., sites) alone only explained 3.9%, indicating that heartwood color traits are largely genetically controlled, whereas environmental differences could be minimal, which could also be suggested by the scanned images of the clones with the highest and lowest L* (Fig 1). The large contribution of genetic effects in the heartwood color of *C*. *japonica* was also reported in a previous study conducted in a single forest stand [28]. These findings indicated that heartwood colors in this species have high potential to be improved through breeding in various environmental conditions, which is particularly important for long-lived forest trees that experience substantial environmental changes such as ongoing climate change.

The effect of environmental differences on heartwood color varied among heartwood color traits. For example, environmental differences only explained 2.4% and 14.6% of the total variance in L* and a*, respectively. These results indicated that the environmental difference could affect heartwood color in terms of reddish hue (red/green) but not the brightness in *C*. *japonica*. As noted above, a* was significantly higher for mapping progenies in the Chiba site, which was the only site without significant spatial autocorrelation in either heartwood color trait. These results suggested that environmental conditions in the Chiba site could have affected the reddish hue of the heartwood of mapping progenies. Although there was not sufficient evidence to evaluate the cause of these results, the soil analysis results implied that pH and potassium content in the soil of the Chiba site was higher and lower, respectively, than the other sites. These soil compositions may have altered the reddish hue of the heartwood of mapping progenies. Indeed, previous studies reported that heartwood color in *C*. *japonica* was influenced by potassium content [33].

The interaction between genotype and environment explained 10.6% on average of the total variance and comprised 26% of the total genetic effects, indicating that genotype-by-environment interaction is important for understanding the genetic basis of heartwood color in *C*. *japonica*. Mori et al. [13] reported that the variablity of heartwood moisture content explained by genotype and genotype-by-environment interaction were 54.8% and 39.6%, respectively, using the same mapping progenies of this study, indicating that the variability explained by genotypes and genotype-by-environment interaction on heartwood moisture content was larger than heartwood color traits. These findings indicated that heartwood color traits are largely genetically controlled, whereas heartwood moisture content is more genetically controlled than heartwood color traits.

### QTLs controlling heartwood color

QTL analysis identified a large number of QTLs for L*, a*, and b*, and most of these QTLs had small to moderate effects (PVE = 7.0% on average) on heartwood color traits. This tendency was consistent with previous studies that identified QTLs for wood properties and growth traits in tree species (reviewed in [1]). The number of identified QTLs differed largely between sites and traits. The largest number of significant QTLs was identified for L*, and the PVE of L* was larger than a* and b*. These indicated that brightness could be more genetically controlled by QTLs than other heartwood traits, such as a* and b*, and implied that brightness could be suitable for molecular breeding (e.g., genomic selection; [44, 45]) of heartwood colors in *C*. *japonica*. In contrast, the largest and smallest numbers of QTLs were identified in Ibaraki and Chiba sites, respectively. As noted above, the soil conditions of the Chiba site could be substantially different compared to other sites, suggesting that the environmental conditions in the Chiba site may have hindered the expression of QTLs.

One QTL identified for b* was detected in the three sites. In addition to this QTL, three QTLs identified for spectral reflectance of short wavelengths (<520 nm) were also detected in the three sites (S7 Table). These QTLs were considered stable to environmental changes. In contrast, most of the identified QTLs were detected uniquely in either site, indicating that QTLs are mostly affected by environments, consistent with a previous study of the mapping progeny [13]. The stable QTLs identified for b* were located within the QTL cluster of 94.0 to 99.1 cM in LG06 of the YI96 map. In addition, seven of eight QTLs identified for L*, a*, and b* located within this QTL cluster were detected in two or more sites, and these QTLs had relatively larger PVE (9.3% on average) than the other QTLs (6.7% on average). These findings suggested that there might be some important genes that control heartwood color traits in a variety of environments near the QTL cluster.

Some QTLs had pleiotropic effects on heartwood color and moisture content. For example, a QTL (m_1540) located in LG09 of the YI38 map was detected in heartwood color traits of L* and spectral reflectance of 400–700 nm (S6 Table) and this QTL was also identified for heartwood moisture content in the same study site ([13]; S6 Table). This might indicate that there could be some important pleiotropic genes controlling both heartwood moisture content and color in *C*. *japonica* near the QTL. This further indicates the importance of identifying QTLs not only for a single trait of interest but also for multiple traits in understanding the genetic basis of the complex traits in forest trees.

## Conclusion

This study evaluated the relative contribution of genetic and environmental effects using multiple common gardens in contrasting environments and identified QTLs controlling heartwood color traits in *C*. *japonica*. Overall, genetic effects, including genotype-by-environment interaction, had a large effect on heartwood color traits (L*a*b* color space and spectral reflectance), whereas environmental effect alone explained less variance. Therefore, not only genetic effects alone but also genetic variation in phenotypic plasticity were considered important for breeding of heartwood color in *C*. *japonica* under changing environments. These findings showed that heartwood color in *C*. *japonica* has high potential and is suitable in terms of improving complex traits through breeding programs in wide range of environmental conditions. In addition, this study found a large number of QTLs with relatively small effects; however, some QTLs were expressed stably under multiple environments or had pleiotropic effects on heartwood color and moisture content. These findings indicated that molecular breeding approaches such as MAS and genomic selection are possible in improvement programs on heartwood color in *C*. *japonica*; MAS may be possible in the improvement programs conducted within a small range of environmental conditions, while further studies on identification of genes controlling heartwood color are essential for successful marker-based improvements. On the other hand, genomic selection would be effective in improvement programs under wide range of environmental conditions. These necessitates further studies with different genomic approaches such as association mapping studies [4, 46, 47] to validate the findings of this study and to capture wider genetic variations because the present study was limited to segregated populations derived from local cultivars.

## Supporting information

S1 Table Heartwood color traits of the three cultivars (Iwao, Yabukuguri, Kumotooshi) obtained from a previous study (Nakada R, et al. 1998 Mokuzai Gakkaishi. 44:395–402). (XLSX)

S2 Table Results of ANOVA for heartwood colors with cultivar, tree (ramet), and measurement side as variables. (XLSX)

S3 Table Results of soil analysis in the three sites. (XLSX)

S4 Table Summary of Moran’s *I* test of heartwood color traits. (XLSX)

S5 Table Summary of the model comparisons of QTL analysis. (XLSX)

S6 Table List of QTLs identified for spectral reflectance. (XLSX)

S7 Table List of stable QTLs for heartwood color traits (L*a*b* color space and spectral reflectance) identified in the three sites. (XLSX)

S8 Table QTLs identified for both heartwood color and property traits. (XLSX)

S9 Table Correlation matrix of heartwood color traits (L*a*b* color space and spectral reflectance) for each site. (XLSX)

S10 Table List of markers used in the present study. (CSV)

S1 Appendix Correlation networks of the heartwood color traits for each sites. (TIF)

S2 Appendix Linkage maps of YI38 with QTLs for heartwood color traits (L*a*b* color space and spectral reflectance). (TIF)

S3 Appendix Linkage maps of YI96 with QTLs for heartwood color traits (L*a*b* color space and spectral reflectance). (TIF)

S4 Appendix Number of QTLs identified for heartwood color traits (L*a*b* and spectral reflectance) for each site. (TIF)
