# Supplementary figures and images for: Genotype-by-environment interaction and genetic dissection of heartwood color in Cryptomeria japonica based on multiple common gardens and quantitative trait loci mapping
Source: PLoS One. 2022 Jul 6;17(7):e0270522. doi: 10.1371/journal.pone.0270522 (PMC9258842; doi:10.1371/journal.pone.0270522)

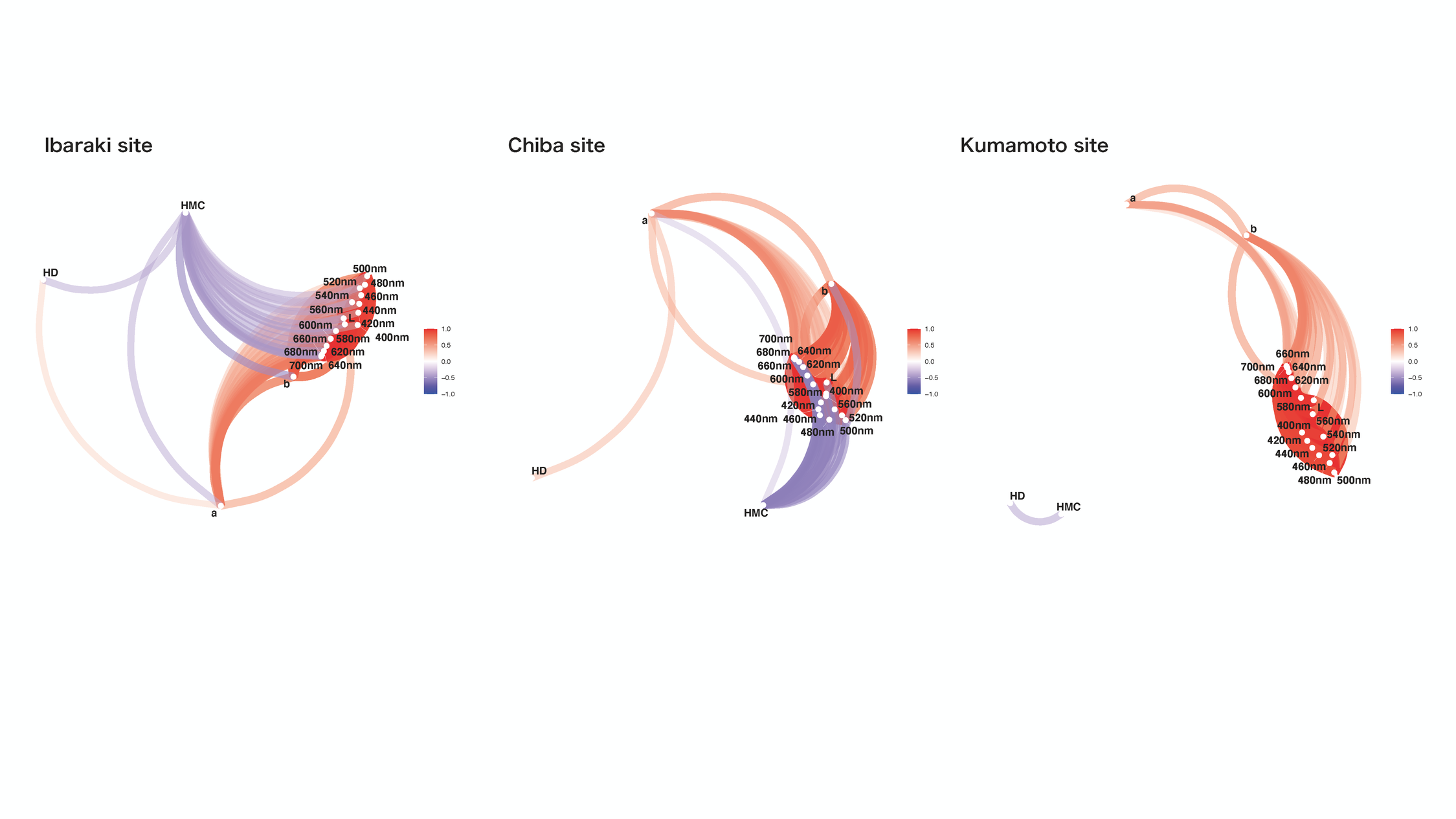

Supplement: S1 Appendix — (TIF) [file pone.0270522.s011.tif]

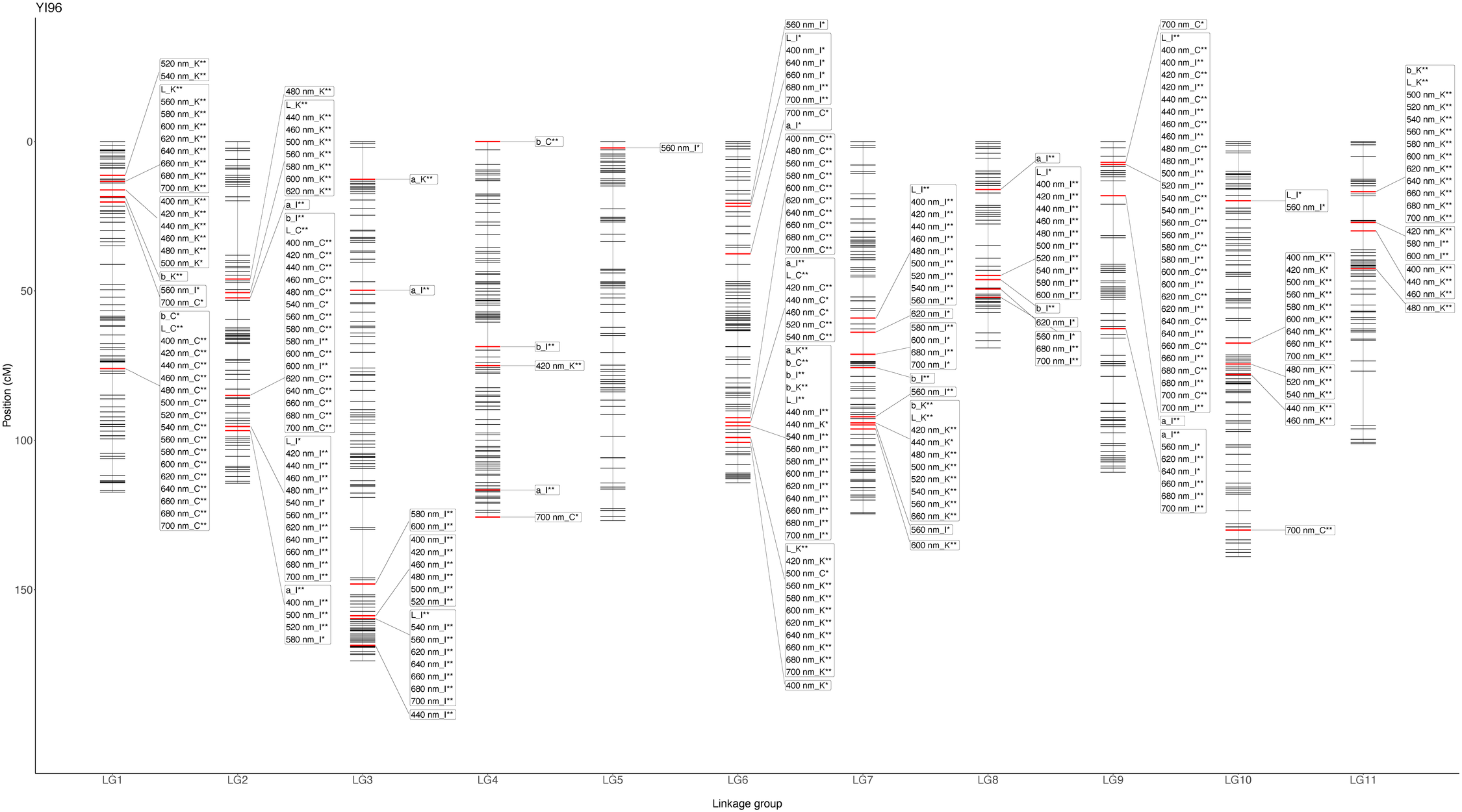

Supplement: S2 Appendix — (TIF) [file pone.0270522.s012.tif]

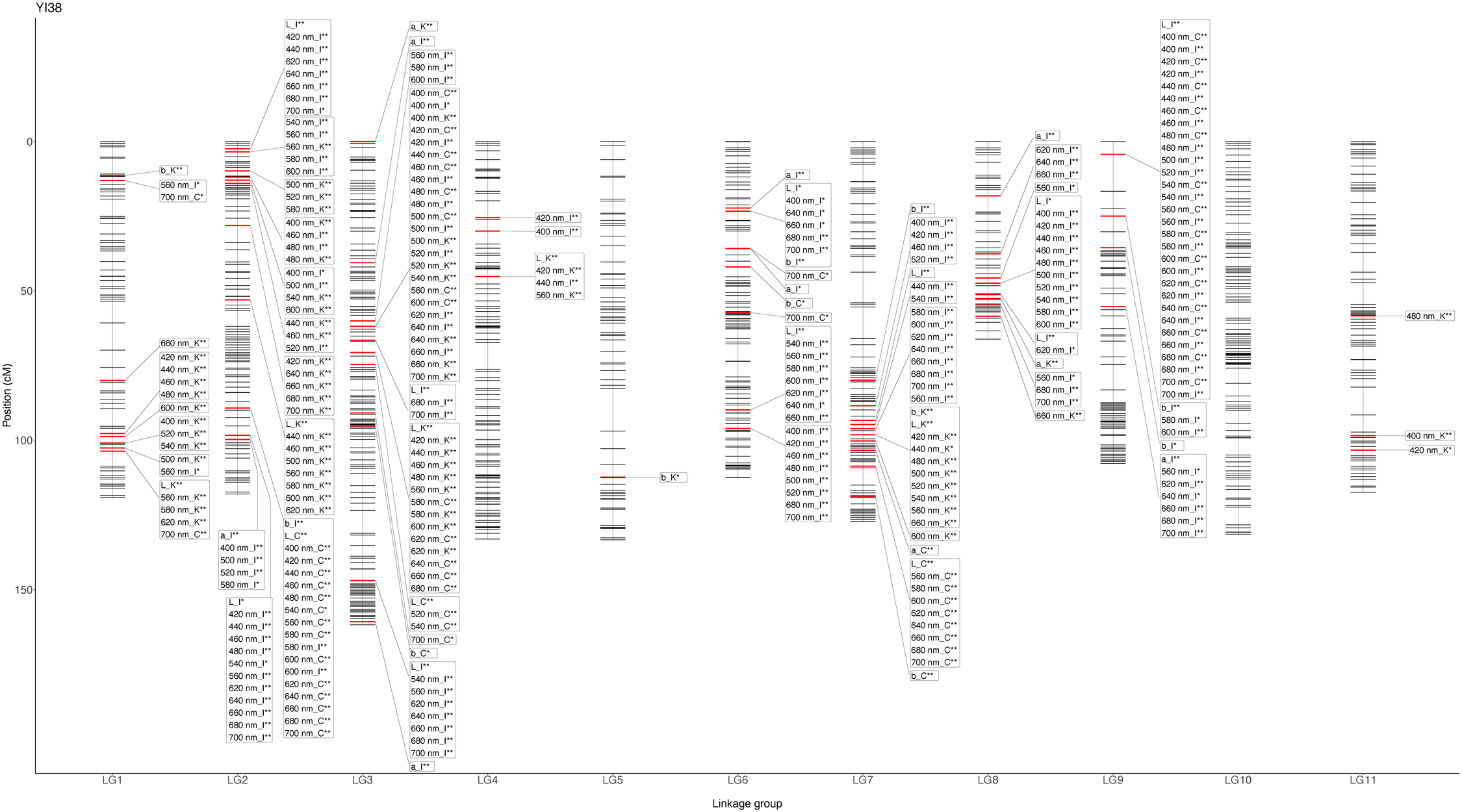

Supplement: S3 Appendix — (TIF) [file pone.0270522.s013.tif]

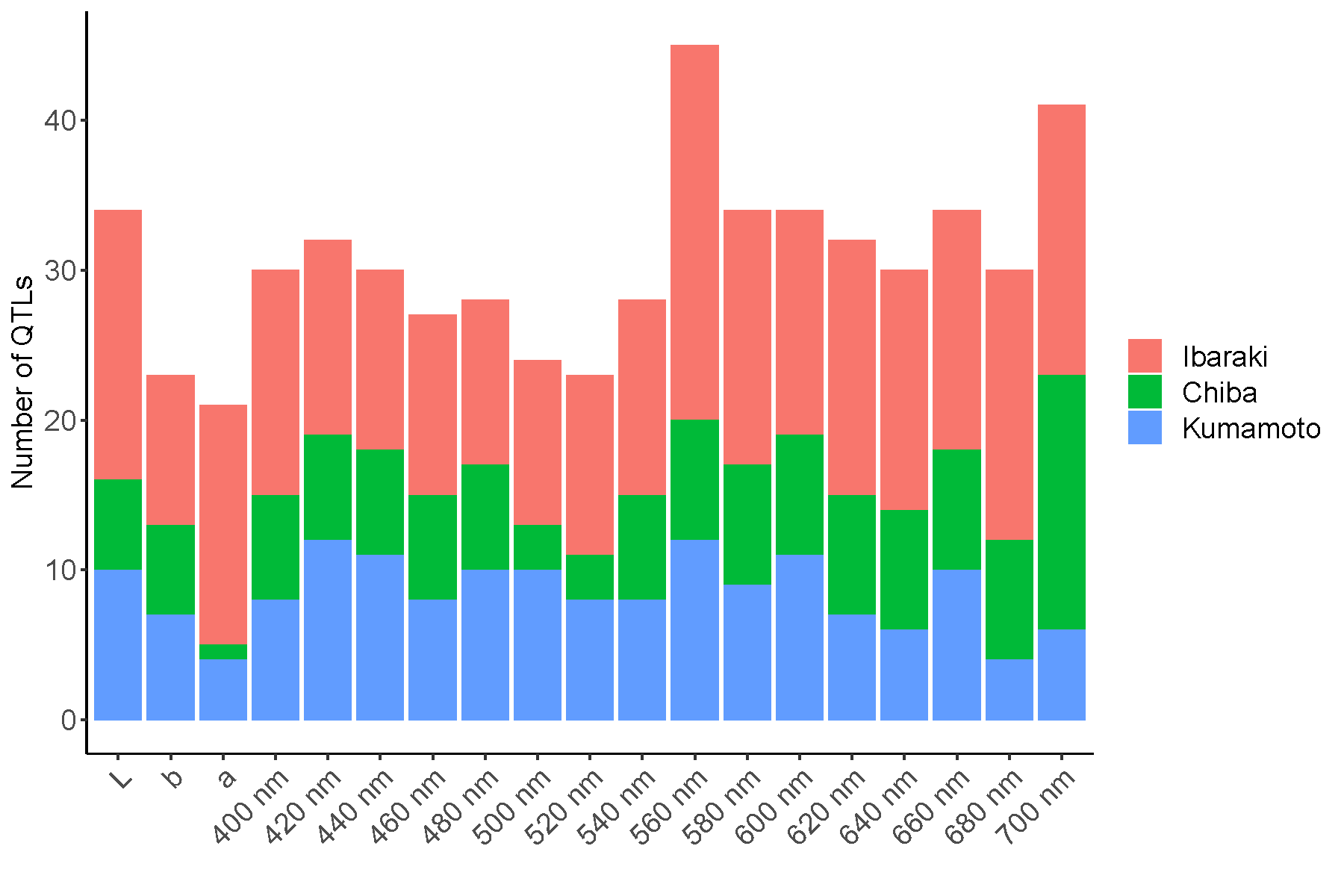

Supplement: S4 Appendix — (TIF) [file pone.0270522.s014.tif]
